# CMMRD caused by *PMS*1 mutation in a sudanese consanguineous family

**DOI:** 10.1186/s13053-022-00222-4

**Published:** 2022-04-15

**Authors:** Reem S. Hamad, Muntaser E. Ibrahim

**Affiliations:** Institute of Endemic Diseases, University of Khartoum, Khartoum, Sudan

**Keywords:** CMMRD, PMS1, Consanguinity, Cancer

## Abstract

A consanguineous family of three siblings presented with different early onset pediatric cancers. Whole-exome sequencing of parents DNA revealed a deleterious frameshift mutation in h*PMS1* the first to be reported in association to a CMMRD phenotype.

## Main text

Germline mutations in Mismatch repair (MMR) genes [1] may result in various hereditary cancer syndromes including Lynch syndrome (LS), Constitutional MMR deficiency (CMMRD) and a recently reported recessive polyposis syndrome-associated with biallelic mutations in MSH3 [2].

While LS caused by pathogenic germline mutations in MLH1, MSH2, MSH6 and PMS2, is known to be characterized by early adult-onset colorectal cancer and an increased risk of other associated tumors [3] CMMRD caused by biallelic mutations in MMR genes particularly features with development of dermatological lesions, hematological, brain and colorectal malignancies, which occur mainly during childhood and adolescence. Although overlapping clinical phenotypes have been described between these syndromes [2–5] common features are seen at the somatic level as well, like microsatellite instability and/or loss of mismatch repair protein expression. Within the MMR genes PMS2 harbors most of the underlying gene defects accounting for approximately 60% of cases [3], while the remaining 40% are equally distributed among other members of the MMR family [4].

PMS1 is a key element in the mismatch repair system and its role in DNA repair is authenticated by studies in hPMS1 knockout mice. These mice despite not developing tumors, displayed poly(A) tract mutation frequencies above normal levels [6]. In addition, *hPMS*1 is found to be down regulated in all microsatellite instability positive tumors in young and older patients [7]. Although hPMS1 was found mutated in the germline of one HNPCC family [5], no PMS1 mutations have been reported so far to be associated with any of the above cancer syndromes.

**Fig. 1:**
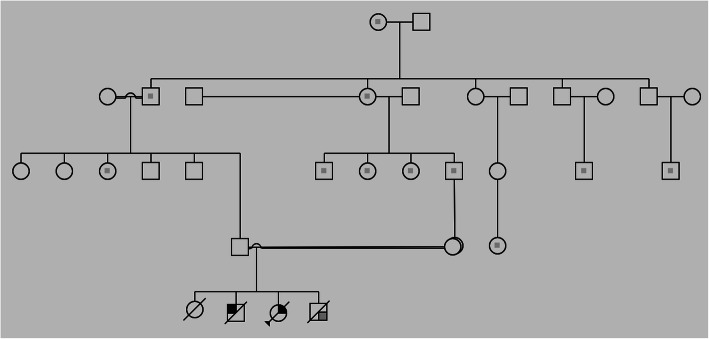
Family pedigree for four generations of the reported cases based on information obtained from family members and oral autopsies as well as basic medical records. Square in the middle indicate a case of colorectal cancer, square in corners: Brain tumors, leukemia and lymphoma

Here we report the first case of CMMRD caused by PMS1 germline mutation in the offspring of an apparently healthy couple from North Sudan. The couple belongs to a consanguineous family, with history of multiple colorectal cancer cases (Fig. 1). They suffered the death of all their four children where 3 siblings presented with different early onset pediatric cancers and subsequently died as a consequence of their malignancies while their eldest child has reportedly succumbed to malaria. Their second child, a male diagnosed at the age of 1.5 years with leukemia died at the age of two. The third is a female diagnosed with lymphoma at the age of 9 and died at the age of 11. The fourth is a male diagnosed with brain tumor and died at the age of six. Due to poor documentation little is known about the type of malignancy and its management.

After their fourth child death, the pediatrician sent the couple to our genetic clinic for consultation. Given the above, we suspected that it is most likely either CMMRD or Li-Fraumeni syndrome and according to the disease scoring criteria we discussed with the parents the option of genetic testing. Whole-exome sequencing was carried out for the parents upon their request. Genomic DNA was sent to BGI, China for WES. A frame shift mutation at PMS1 variant has been found (NM_001321049.1:p.Leu164fs/c.488dupA). The mutation is in exon 5 with an extra Adenine at a microsatellite area of Adenine. Both parents were carriers for this mutation.

It is essential to determine the molecular cause of CMMRD to allow for predictive testing in at-risk relatives. Individuals with mono-allelic MMR mutation in these families should follow LS screening recommendations. The identification of these inherited conditions has important consequences for clinical management, allowing targeted preventive measures in mutation carriers. In this family we encountered a novel mutation, the first PMS1 to be reported in association to CMMRD syndrome, expanding our perspective about the possible genetic causes of this syndrome.

The community in North Sudan to which the family belongs is one that is notorious for its culture of consanguineous marriage as seen in the family pedigree. Cultures of consanguinity and within group marriage are confined to certain ethnic groups in Sudan and are known to be major cause of increased load of genetic disease in these communities. It is obviously also a lever for the emergence of genetic disorders caused by low frequency or rare germ line mutations as in the current case of CMMRD. The segregation of these alleles seems contra intuitive to its recessive nature and a large African effective population size with multiplicity of pathogenic minor alleles. However, this could be readily explained by the effect of consanguinity a cultural practice that acts on decreasing genetic diversity and increasing the chances of allele segregation. We have shown the relevance of such caveat particularly in the challenge of identification and culpability of pathogenic variants [8].

In all cases Exome and whole genome sequencing remains the most convenient tools for diagnosing Mendelian and rare genetic disorders including cancer particularly in the unique African setting.

## Data Availability

The datasets used and/or analyzed during the current study are available from the corresponding author upon reasonable request.

## Abbreviations

LS: Lynch Syndrome
HNPCC: Hereditary non polyposis colorectal cancer
